# hucMSCs Attenuate IBD through Releasing miR148b-5p to Inhibit the Expression of 15-lox-1 in Macrophages

**DOI:** 10.1155/2019/6953963

**Published:** 2019-05-28

**Authors:** Jingjing Kang, Zhaoyang Zhang, Jingyan Wang, Gaoying Wang, Yongmin Yan, Hui Qian, Xu Zhang, Wenrong Xu, Fei Mao

**Affiliations:** ^1^Key Laboratory of Medical Science and Laboratory Medicine of Jiangsu Province, School of Medicine, Jiangsu University, Zhenjiang, Jiangsu 212013, China; ^2^Taicang Hospital of Traditional Chinese Medicine, Suzhou, Jiangsu 215400, China

## Abstract

Mesenchymal stem cells (MSCs) exert powerful immunosuppression in inflammatory bowel disease (IBD). Macrophages are the dominant inflammatory cells in enteritis regulated via MSCs. However, the roles of macrophages in the process of MSCs attenuating IBD and the mechanisms of MSCs regulating macrophages are largely unknown. In this study, DSS- (dextran sulfate sodium salt-) induced IBD in macrophage-depleted models of CD11b-DTR mice was used to study the relationship between hucMSCs (human umbilical cord mesenchymal stromal cells) and macrophage. Body weights, disease activities, and pathological changes were documented to assess the therapeutic effects of hucMSCs. Furthermore, hucMSCs transfected with miR148b-5p mimics and miR148b-5p inhibitors were cocultured with LPS-induced RAW264.7 cells to investigate the role of miR148b-5p in hucMSC-regulated colitis. The outcome indicated that hucMSCs attenuated the IBD by downregulating 15-lox-1 expression in macrophages. Further findings pointed out that hucMSCs transfected with miR148b-5p mimics could be elevated to promote the tissue repair and inhibit the expression of 15-lox-1 but failed to perform the function of easing enteritis when treated with miR148b-5p inhibitors. In conclusions, we propose that hucMSCs attenuate IBD by releasing miR148b-5p to inhibit the expression of 15-lox-1 in macrophages.

## 1. Introduction

Inflammatory bowel disease (IBD) containing ulcerative colitis (UC) and Crohn's disease (CD) is characterized by idiopathic mucosal inflammation involving the entire gastrointestinal mucosa [1]. The most basic pathogenesis pattern of either UC or CD consists in the excessive activation of innate and adaptive immune responses and the release of inflammatory factors activated via cells like inflammatory T effector cells and macrophages [2]. The incidence of IBD in Asia is gradually elevating and closely follows the trend of Western countries [3, 4]. Traditional therapy for IBD mainly consists of immunosuppressive therapy [5], monoclonal antibody therapy [6], and surgery [7]. The aforementioned therapeutics cannot fully meet the demands of clinical treatment for their trauma or the inability to fundamentally reverse excessive immunity [8]. With the increase in the prevalence of IBD, it is urgent to seek for a therapeutic option to improve existing strategies and alleviate patients' suffering.

Mesenchymal stem cell- (MSC-) based therapy for the treatment of IBD is novel and promising for its advantages of low immunogenicity and immunosuppression [9]. Moreover, MSCs can be induced to differentiate into adipocytes, chondrocytes, neural cells, etc. to exert the function of tissue repair [10]. With these characteristics, MSC therapy of IBD is aimed not only at inhibiting mucosal inflammation but also at repairing the damaged mucosa and promoting the mucosal tissue regeneration [11]. The combination of cell-cell communication and paracrine pathway contributes to the powerful immunosuppression of MSCs [12]. As shown in previous studies, MSCs can suppress the activation of T-helper (Th)1 cells and Th17 cells and the promotion of T regulatory (Treg) cell multiplication mainly due to the paracrine factors released by MSCs, possessing a large number of bioactive proteins and miRNAs [13, 14]. Simultaneously, MSCs are also able to control the polarization of macrophages [15] and the excitation of other antigen-presenting cells [16]. However, the role of macrophages in the process of MSCs alleviating enteritis is unknown and the mechanism of MSCs regulating macrophages to suppress inflammation is still uncovered. It is reported that 15-lox-1 is a crucial moderator of inflammatory response in the colon and other tissues and it is mainly expressed in the macrophages [17].

In this study, a DSS-induced IBD mouse model was adopted to evaluate the mechanism of hucMSCs on the repair of IBD and the CD11b-DTR mice were used to achieve macrophage depletion on the basis of previous studies [18].

## 2. Methods

The study was approved by the Ethical Committee of Jiangsu University (2012258).

### 2.1. Cell Culture

hucMSCs were isolated as previously described and cultured in MEM *α* medium (Invitrogen) [19]. RAW 264.7 cells were cultured in RPMI 1640 medium (Invitrogen). They were maintained in medium containing 10% (*v*/*v*) fetal calf serum at 37°C in humid air with 5% CO_2_.

### 2.2. Animal Model Establishment and Treatment

CD11b-DTR mice (6 weeks old) were purchased from the Nanjing Biomedical Research Institute of Nanjing University (Jiangsu, China). Bab/c mice (6 weeks old) were purchased from the Laboratory Animal Research Center of Jiangsu University (Jiangsu, China). All experimental procedures were conducted in accordance with the Animal Use and Care Committee of Jiangsu University.

The mice were divided into different groups (*n* = 6/group) according to different objectives. Mice were exposed to 3% (*w*/*v*) dextran sulfate sodium (DSS) in the drinking water for 10 days. On days 3, 6, and 9, PBS or hucMSCs (3 × 10^6^) were injected intraperitoneally (i.p.) according to the experimental prescription of previous research [20]. Recombinant adenovirus of 15-lox-1 or GFP (1 × 10^9^ PFU) (Thermo Fisher Scientific) were injected intravenously (i.v.) two days before the experiment began. Mice were weighed, and their stools monitored daily to measure the weight loss and disease activity index (DAI) [21]. All mice were sacrificed at days 9, 10, or 11; their colons and spleens tissues were collected; and the splenic mononuclear cells and colon mucosa were used for further studies.

### 2.3. Macrophage Depletion in CD11b-DTR Mice

Diphtheria toxin (DT, unnicked) (Cayman) was administered i.p. in CD11b-DTR mice at a concentration of 25 ng/g every 4 days. On the second day of DT injection, the peritoneal macrophages (2 × 10^6^) extracted from CD11b-DTR mice were stained with CD11b^+^ monoclonal antibody (PE; 1 : 200; eBioscience) and F4/80^+^ monoclonal antibody (APC; 1 : 200; eBioscience) for 30 minutes at 4°C. Flow cytometry (FCM) was performed to analyze the ratio of CD11b^+^ F4/80^+^ cells to verify the macrophage depletion rate.

### 2.4. Immunofluorescence Analysis

RAW 264.7 cells were fixed in 4% paraformaldehyde for 30 minutes, permeabilized for 5 minutes with 0.5% Triton-X100, blocked with 5% bovine serum albumin, and incubated with rabbit monoclonal anti-F4/80 and anti-CD206 (1 : 200; Santa Cruz) antibody overnight at 4°C, followed by incubation with diluted secondary antibody at 37°C for 60 minutes. The nuclei were counterstained with Hoechst 33342 (1 : 300; Sigma-Aldrich). Images were acquired sequentially with a superresolution fluorescence microscopy (Nikon, Tokyo, Japan, https://www.nikon.com).

### 2.5. Immunohistochemistry Analysis

Formalin-fixed paraffin-embedded colon and spleen tissues of mouse (4 *μ*m thick) were stained by hematoxylin-eosin (HE) or were dewaxed for immunohistochemistry. Endogenous peroxidase activity was then inhibited by exposure to 3% hydrogen peroxide for 30 min, and antigen retrieval was realized through boiling for 30 min in citrate buffer (pH 6.0, 10 mM). The sections were then blocked with 5% BSA and incubated with 15-lox-1 (1 : 200; Abcam), F4/80 (1 : 200; Santa Cruz), and PCNA (1 : 200; Cell Signaling Technology) primary antibody overnight at 4°C. Finally, sections were visualized using diaminobenzidine (DAB) substrate and counterstained with hematoxylin for microscopic examination.

### 2.6. RNA Extraction and Quantitative Real-Time PCR

The RNA was extracted from the colon mucosa or splenic mononuclear cells, hucMSCs, and RAW 264.7 cells using Trizol Reagent (Life Technologies, Carlsbad, CA, USA). The cDNAs were synthesized by using the HiScript 1st Strand cDNA Synthesis Kit (Vazyme Biotech, Shanghai, China) and miScript II RT Kit (QIAGEN). miRNA quantification was determined by using miDETECT A Track™ miRNA qRT-PCR Primer Set specific for miR-148b-5p, designed by RiboBio (RiboBio Co. Ltd., Guangzhou, Guangdong, China). Quantitative real-time polymerase chain reaction (QRTPCR) was carried out in a Step One Plus Real-Time PCR System (Applied Biosystems, Life Technologies, USA) to detect the expression of IL-1*β*, TNF-*α*, IL-6, 15-lox-1, and miR148b-5p. The sequences of specific primers are listed in Table 1.

### 2.7. Western Blot

The colon mucosa or splenic mononuclear cells, hucMSCs, and RAW 264.7 cells were homogenized and modified in RIPA lysis buffer, added with proteinase inhibitors (Vazyme Biotech, Shanghai, China). Protein samples (two hundred micrograms) were separated on a 12% SDS-PAGE (sodium dodecyl sulfate-polyacrylamide gel electrophoresis). Sources and dilution factors of primary antibodies were the following: anti-caspase-3 (1 : 800; Bioworld), anti-PCNA (1 : 1000; Cell Signaling Technology), anti-15-lox-1 (1 : 1000; Abcam), and anti-*β*-actin (1 : 800; Bioworld). After incubation with the primary antibodies overnight at 4°C, the blots were incubated with the secondary antibodies for 1 h at room temperature and then were visualized by chemiluminescence (Millipore, USA) and detected by using the imaging software (GE Healthcare, Life Sciences, USA).

### 2.8. miR148b-5p Mimics and Inhibitor Transfection

miR148b-5p mimics, inhibitor, negative mimic control, and negative inhibitor control were purchased from Gene Pharma. Negative mimic control and negative inhibitor control were mixed to be identified as the control group. Mimics or negative control at a final concentration of 50 nM and inhibitor at a final concentration of 100 nM were mixed with Lipofectamine 2000 (Gene Pharma) according to the manufacturer's instructions and transfected into hucMSCs cultured to 70%–80% confluence in a culture dish.

### 2.9. Coculture of RAW 264.7 with hucMSCs

RAW 264.7 cells (10^5^) were seeded on 6-well culture plates, which were stimulated with lipopolysaccharide (LPS) (Sigma-Aldrich; 100 ng/ml), and co-cultured with hucMSCs (5 × 10^4^) in a transwell system (Corning; 0.4 *μ*m) for 48 h.

### 2.10. Luciferase Reporter Assay of miRNA Target

The 3′-UTR regions of the 15-lox-1 mRNA containing the predicted binding sites for miR-148b-5p, wild or mutant (AGAACTA mutated to ACAAGAA), were cloned into pmirGLO Dual-Luciferase miRNA Target Expression Vector (GUR100509 and GUR100510; RiboBio). Negative mimic control and negative inhibitor control were mixed to be identified as the control group. After cotransfection with the vectors and miR-148b-5p mimic, miR-148b-5p inhibitor, or control, the firefly and Renilla luciferase activities were measured using the Dual-luciferase Reporter Assay (Promega, Madison, WI, USA).

### 2.11. Statistical Analysis

All data were shown as the means ± SEM. Statistical analysis was performed by Student's *t*-test or by analysis of variance (ANOVA) using Prism software (GraphPad, San Diego, CA). *P* < 0.05 was considered statistically significant.

## 3. Results

### 3.1. Macrophages Are involved in DSS-Induced IBD

**Figure 1:**
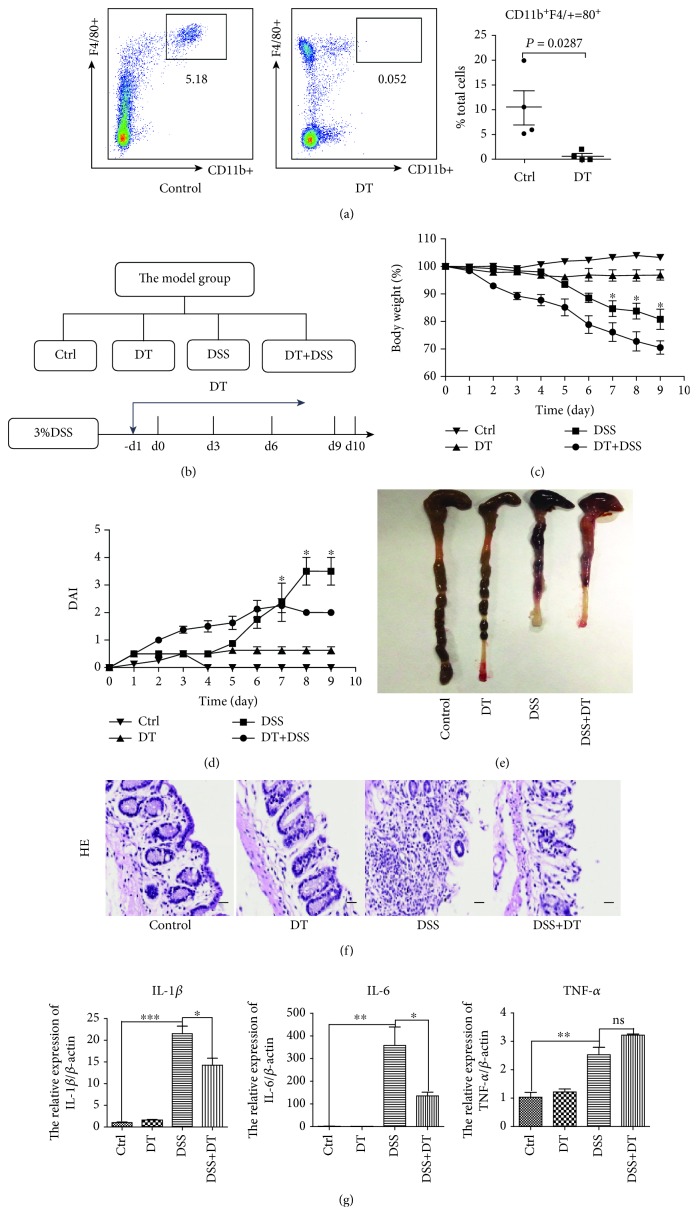
Macrophages are involved in DSS-induced IBD. (a) FCM was applied to identify the peritoneal macrophage depletion. (b) The model group was designed to contain the control, DT, DSS, and DT+DSS groups. DT was given 1 day prior to DSS treatment. (c) The body weight loss and (d) the disease activity index (DAI) of each group are presented. (e) The colon lengths of each group are presented. (f) Hematoxylin and eosin (HE) staining of colon tissues of each group is presented (100x, scale bar = 100 *μ*m). (g) The inflammatory cytokine (IL-1*β*, IL-6, and TNF-*α*) expression of colon tissues was measured via QRT-PCR analyses. *N* ≥ 6 for each group. Data shown were representative of three independent experiments. Data represent the mean ± SEM. ^∗^ *P* < 0.05, ^∗∗^ *P* < 0.01, and ^∗∗∗^ *P* < 0.001 by ANOVA.

Our previous study found that hucMSCs can relieve enteritis and inhibit the expression of F4/80, a marker of macrophages [20]. To determine the role of macrophages in the development of IBD, we procured CD11b-DTR mice and depleted them of macrophages using the administration of DT 1 day prior to DSS treatment. The flow cytometric analysis of peritoneal cells showed that administration of DT resulted in a nearly complete depletion of CD11b^+^F4/80^+^ cells, which represent myeloid-derived macrophages (Figure 1(a)). To investigate whether macrophages were involved in IBD, CD11b-DTR mice were divided into four groups as follows: control, DT, DSS, and DT+DSS groups (Figure 1(b)). The results showed that macrophage depletion exacerbated the weight loss (Figure 1(c)) and reduced the DAI (Figure 1(d)) compared with mice treated with only DSS. The average colon length in the DT+DSS group had no significant difference from that in the DSS group, but the average size of the spleens of the DT+DSS group was smaller than that of the DSS group (Figure 1(e) and Figure S1(a)). The structural integrity of colon tissues was repaired, and the splenic nodules were rebuilt to a certain extent in the DT+DSS group compared with that in the DSS group (Figure 1(f) and Figure S1(b)). As shown in Figure 1(g) and Figure S1(c), the expression of inflammatory factors (IL-1*β*, TNF-*α*, and IL-6) in the colon and spleen tissues of the DT+DSS group was lower than that of the DSS group. Therefore, we can infer that after the depletion of macrophages, although inflammation still exists, DSS-induced enteritis becomes significantly alleviated. In summary, these results indicate that macrophages are involved in DSS-induced IBD.

### 3.2. hucMSCs Attenuate the DSS-Induced IBD through Regulating Macrophages

The above results suggest that macrophages are involved in DSS-induced enteritis. Other studies have also reported that MSCs can regulate a variety of immune cells to play immunosuppressive functions, including macrophages, but its mechanism is unknown. First, the hucMSCs used in the experiment were identified. Flow cytometry results showed that CD73, CD90, and CD105 were positive on the surface of hucMSCs, but CD34, CD45 and HLA-DR were negative (Figure S2(a)). After the induction of osteogenic and adipogenic differentiation culture using selective medium, oil-red-O staining showed that hucMSCs formed lipid droplets (Figure S2(b)) and Alizarin Red staining proved that hucMSCs could exhibit calcium deposition (Figure S2(c)). The above results indicated that the cells used in the experiments accord with the phenotypic characteristics of hucMSCs.

**Figure 2:**
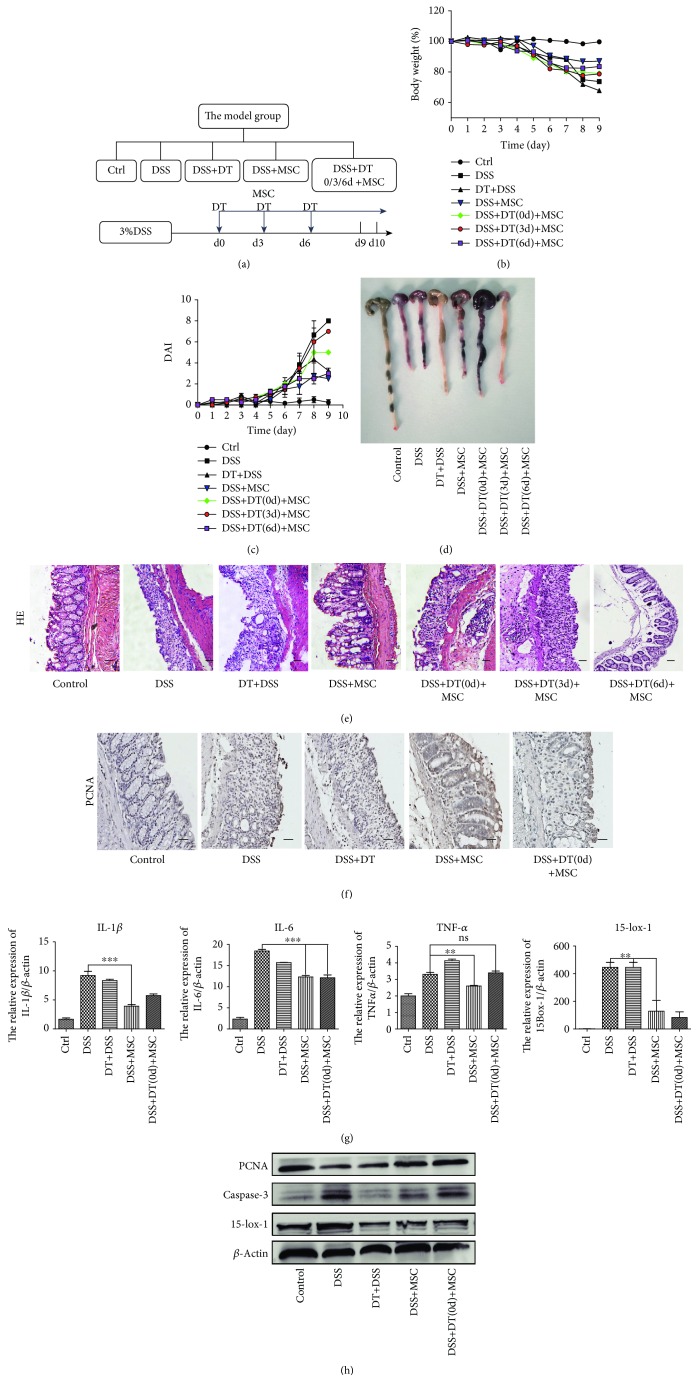
hucMSCs attenuate the DSS-induced IBD through regulating macrophages. (a) The model group was designed to contain the control, DSS, DT+DSS, DSS+MSC, and DSS+MSC+DT (days 0, 3, and 6) groups. DT was given at days 0, 3, and 6. (b) The body weight loss and (c) the DAI of each group are presented. (d) The colon lengths of each group are presented. (e) HE of colon tissues of each group is presented (100x, scale bar = 100 *μ*m). (f) IHC of PCNA expression in the colon tissues of each group are presented (100x, scale bar = 100 *μ*m). (g) The inflammatory cytokines (IL-1*β*, IL-6, and TNF-*α*) and 15-lox-1 expression of colon tissues were measured via QRT-PCR analyses. (h) The expression of PCNA, caspase-3, 15-lox-1, and *β*-actin proteins in the colon tissues was measured by Western blot. *N* ≥ 6 for each group. Data shown were representative of three independent experiments. Data represent the mean ± SEM. ^∗^ *P* < 0.05, ^∗∗^ *P* < 0.01, and ^∗∗∗^ *P* < 0.001 by ANOVA.

In order to determine whether hucMSCs attenuate the DSS-induced IBD by regulating macrophage, the model group was designed to contain the control, DSS, DSS+DT, DSS+MSC, and DSS+MSC+DT groups. DT was given at days 0, 3, and 6 while hucMSCs were administered i.p. at days 3, 6, and 9 (Figure 2(a)). Various indicators were measured to determine whether the role of hucMSCs in repairing IBD is disrupted after macrophage depletion. The weight loss and DAI differences between DSS+MSC+DT (day 0) and DSS+MSC+DT (day 3) were not obvious but were more severe compared to those between DSS+MSC and DSS+MSC+DT (day 6) mice (Figures 2(b) and 2(c)). In the colon and spleen of mice, the same trend existed in the general view and microstructure of tissues. The colon length in the DSS+MSC+DT (day 0) group was smaller and hemorrhagic while that in the DSS+MSC+DT (day 3) group was more severe than that in the DSS+MSC and DSS+MSC+DT (day 6) groups. The size of the spleen in the DSS+MSC+DT (day 0) and DSS+MSC+DT (day 3) groups was larger than that in the DSS+MSC and DSS+MSC+DT (day 6) groups (Figure 2(d) and Figure S3(a)). The structural integrity of the colon was destroyed, and the splenic nodules were broken in the DSS+MSC+DT (day 0) and DSS+MSC+DT (day 3) groups. There was no obvious difference in tissue repair between DSS+MSC and DSS+MSC+DT (day 6) (Figure 2(e) and Figure S3(b)). The above data reflect that, when macrophages are removed before or on the day of injection of hucMSCs, the effect of hucMSCs on relieving enteritis will significantly be impaired. In other words, hucMSCs' repairing capability will decline without macrophage in DSS-induced IBD.

The study again analyzed tissue differences between the five groups of mice to further establish the role of macrophages in IBD. Immunohistochemistry (IHC) reflected that the DSS+MSC group had more PCNA-positive cells in the colon and spleen tissues than the DSS+MSC+DT (day 0) group (Figure 2(f) and Figure S3(c)). However, QRT-PCR analyses revealed that the expression of the inflammatory factor (IL-1*β*, TNF-*α*, and IL-6) in the colon and spleen tissues in the DSS+MSC group was lower than that in the DSS+MSC+DT (day 0) group (Figure 2(g) and Figure S3(d)). The administration of both DT and hucMSCs failed to suppress the expression of caspase-3 protein or to boost the expression of PCNA protein compared with that in the DSS+MSC group (Figure 2(h) and Figure S3(e)). This outcome implies that hucMSCs could not inhibit the apoptosis of inflammatory cells or promote the proliferation of tissue cells in the DSS+MSC+DT (day 0) group. In essence, the absence of macrophages declines the ability of hucMSCs to repair DSS-induced IBD. In other words, hucMSCs attenuate DSS-induced IBD through regulating macrophages.

### 3.3. hucMSCs Attenuate the IBD through Regulating 15-lox-1 Expression in Macrophages

**Figure 3:**
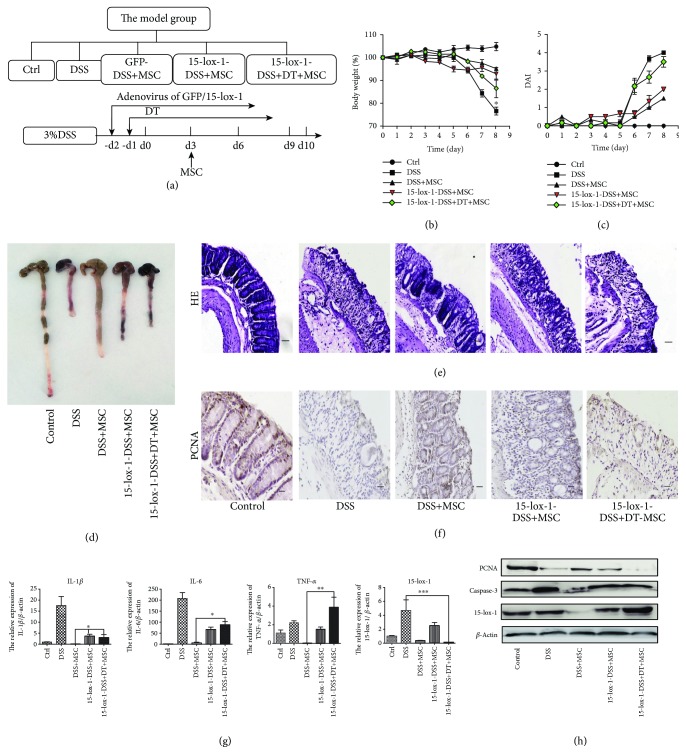
hucMSCs attenuate the IBD through regulating 15-lox-1 expression in macrophages. (a) The model group was designed to contain the control, DSS, GFP-DSS+MSC, 15-lox-1-DSS+MSC, and 15-lox-1-DSS+DT+MSC groups. Adenovirus of GFP/15-lox-1 was given 2 days prior to DSS treatment, and DT was given 1 day prior to DSS treatment. (b) The body weight loss and (c) the DAI of each group are presented. (d) The colon lengths of each group are presented. (e) HE of colon tissues of each group is presented (100x, scale bar = 100 *μ*m). (f) IHC of PCNA expression in the colon tissues of each group is presented (100x, scale bar = 100 *μ*m). (g) The inflammatory cytokines (IL-1*β*, IL-6, and TNF-*α*) and 15-lox-1 expression of colon tissues were measured via QRT-PCR analyses. (h) The expression of PCNA, caspase-3, 15-lox-1 and *β*-actin proteins in the col8on tissues was measured by Western blot. *N* ≥ 6 for each group. Data shown were representative of three independent experiments. Data represent mean ± SEM. ^∗^ *P* < 0.05, ^∗∗^ *P* < 0.01, and ^∗∗∗^ *P* < 0.001 by ANOVA.

It can be inferred from Figures 2(g) and 2(h) that the expression of 15-lox-1 is enhanced but reversed when treated with hucMSCs in IBD mice. Fei et al. had reported that 15-lox-1 was mainly expressed in the macrophages [20]. So in order to verify the interactions between macrophages and 15-lox-1 in the process of hucMSCs alleviating IBD, we designed the following groups: control, DSS, GFP-DSS+MSC, 15-lox-1-DSS+MSC, and 15-lox-1-DSS+DT+MSC groups (Figure 3(a)). The recombinant adenovirus of 15-lox-1 was injected intravenously 2 days prior to DSS treatment to promote the expression of 15-lox-1 in IBD mice, with the recombinant adenovirus of GFP as control, and DT was given 1 day prior to DSS treatment. We then examined the indicators to clarify the role of macrophage 15-lox-1 in hucMSC remission of enteritis. The weight loss, DAI, and tissue injury of the colon and spleen in the 15-lox-1-DSS+MSC group were quite noticeable than those in the GFP-DSS+MSC group, but the differences were not significant. These results reflected that hucMSCs still perform certain repair functions in IBD mice which express 15-lox-1, which may be because hucMSCs regulate 15-lox-1 on macrophages (Figures 3(b)–3(e) and Figure S4(a)-(b)). Again, the weight loss and DAI of the 15-lox-1-DSS+DT+MSC group were more obvious compared with those of the GFP-DSS+MSC and 15-lox-1-DSS+MSC groups. (Figures 3(b) and 3(c)). Additionally, in the 15-lox-1-DSS+DT+MSC group, hucMSCs failed to lengthen the colon or dwindle the size of the spleen and reconstruct the gross and microscopic structure of the colon and spleen compared to mice in the other two groups treated with hucMSCs (Figures 3(d) and 3(e) and Figure S4(a)-(b)). Despite the high expression of 15-lox-1 in mice, hucMSCs were unable to regulate macrophage 15-lox-1, due to their depletion hence the failure to alleviate enteritis. We can therefore infer that hucMSCs alleviate enteritis by regulating the expression of macrophage 15-lox-1.

We further implemented the following experiments to verify our findings. IHC revealed that hucMSCs lost the ability to promote the proliferation of the colon and spleen tissue cells in the 15-lox-1-DSS+DT+MSC group, and QRTPCR exhibited that the expression level of the inflammatory factor (IL-1*β*, TNF-*α*, and IL-6) in the colon and spleen tissues increased in the 15-lox-1-DSS+DT+MSC group compared to the DSS+GFP+MSC group. Western blot also showed that PCNA proteins were inhibited and caspase-3 proteins were promoted in the 15-lox-1-DSS+DT+MSC group (Figures 3(f)–3(h) and Figure S4(c)-(d)). The expression ratio of 15-lox-1 in the colon and spleen tissues was higher in the DSS+15-lox-1+MSC group than that in the control or DSS+GFP+MSC group, indicating that IBD mice that continuously expressed 15-lox-1 were successfully established (Figures 3(g) and 3(h) and Figure S4(d)-(e)). These results show that in IBD mice expressing 15-lox-1, the function of inflammation suppression would be impaired when hucMSCs are unable to regulate macrophage-expressed 15-lox-1. Consequently, Figure 3 indicates that hucMSCs regulate the 15-lox-1 expression in macrophages to attenuate the IBD.

### 3.4. miR148b-5p from hucMSCs Attenuates the IBD through Downregulated 15-lox-1 Expression In Vitro

**Figure 4:**
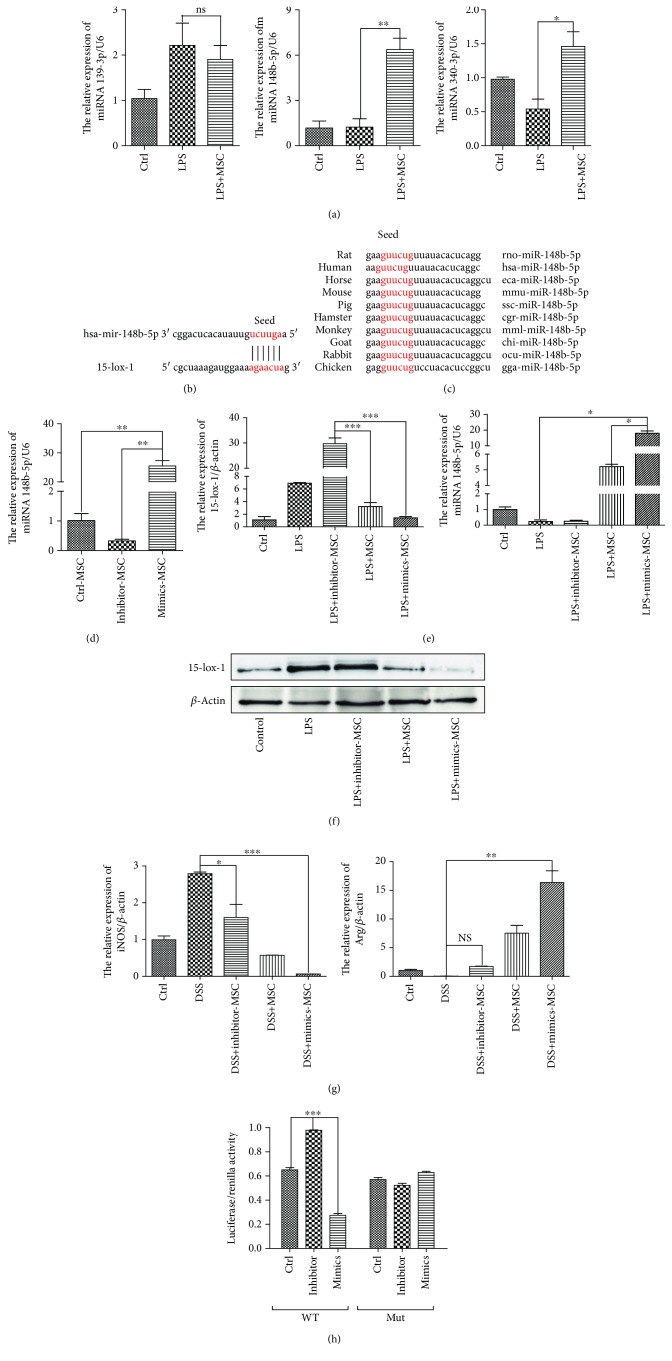
miR148b-5p from hucMSCs attenuates the IBD through downregulated 15-lox-1 expression *in vitro*. RAW 264.7 cells, stimulated with LPS, were cocultured with hucMSCs in a transwell system for 48 h. (a) The miR139-3p, miR148b-5p, miR340-3p, and15-lox-1 expression of RAW 264.7 cells was measured via QRT-PCR analyses. (b) Binding sites between miR148b-5p and 3′UTR of 15-lox-1 mRNA were predicted using miRBase. (c) The sequence of miR148b-5p of different species. (d) hucMSCs were transfected with the miR148b-5p inhibitor, miR148b-5p mimics, or negative control for 48 h. The miR148b-5p expression of hucMSCs was measured via QRT-PCR analyses. RAW 264.7 cells, stimulated with LPS, were cocultured with hucMSCs transfected with the miR148b-5p inhibitor, miR148b-5p mimics, or negative control for 48 h. (e) The miR148b-5p and 15-lox-1 expression of RAW 264.7 cells was measured via QRT-PCR analyses. (f) The expression of 15-lox-1 and *β*-actin proteins in the RAW 264.7 cells was measured by Western blot. (g) The expression of iNOS and Arg-1 in RAW 264.7 cells was measured via QRT-PCR analyses. (h) Luciferase reporter plasmid and miR148b-5p mimics, inhibitor, or negative control were cotransfected with hucMSCs for 48 h, and the reporter luciferase activities were measured. Data shown were representative of three independent experiments. Data represent the mean ± SEM. ^∗^ *P* < 0.05, ^∗∗^ *P* < 0.01, and ^∗∗∗^ *P* < 0.001 by ANOVA.

We further investigated which particular molecule derived from hucMSCs participates in regulating 15-lox-1 to promote relief of IBD. As shown in previous research, the strong immunosuppressive effect of MSCs in IBD was largely due to paracrine function and the exosomes derived from MSCs have been recognized to be involved in major paracrine interactions [14]. Recently, some scholars have found that BMMSC-derived exosomes contain more than 300 miRNAs, which are noncoding small RNAs that direct the silencing complex (RISC) to degrade mRNA or hinder its translation by base pairing with the target gene mRNA [22]. Therefore, we performed comprehensive miRNA profiling in exosomes, derived from hucMSCs or human fibroblast cells (HFL), by using miRNA microarray. Meanwhile, we carried out the prediction of miRNAs silencing the 3′UTR of 15-lox-1 mRNA through TargetScan web server (http://www.targetscan.org). Then, the repeated miRNAs, cross-checked from both results, were reviewed through the existing literature in PubMed database to screen out miRNAs regulating macrophages (https://www.ncbi.nlm.nih.gov/pubmed). Finally, we obtained 3 miRNAs: miR-139-3p, miR-148b-5p, and miR-340-5p. Notably, miR148b-5p is a kind of miRNA that only existed in exosomes derived from hucMSCs but not from HFL. To determine the role of miR148b-5p in the process of hucMSCs regulating 15-lox-1 expression, RAW 264.7 cells, stimulated with LPS to activate IBD environment, were cocultured with hucMSCs in a transwell system for 48 h. RAW 264.7 cells are a kind of commonly used primary macrophage in inflammatory cell models [23]. QRT-PCR analyses revealed that the expression of miR148b-5p was significantly higher than that of miR-139-3p and miR-340-5p in RAW 264.7 cells cocultured with hucMSCs (Figure 4(a)). Thus, miR148b-5p was chosen as the follow-up research object. Binding sites between miR148b-5p and 3′UTR of 15-lox-1 mRNA were predicted using miRBase (Figure 4(b)). The sequence of miR148b-5p of different species showed that miR148b-5p was conservative across different species (Figure 4(c)).

To confirm the prediction, we performed gain- and loss-of-function assay using synthetic oligonucleotides that mimicked miR148b-5p (mimics) or were complementary to miR148b-5p (inhibitor). hucMSCs were transfected with miR148b-5p mimics or inhibitor for 48 h, and then QRT-PCR analyses confirmed that miR148b-5p mimics or inhibitors were highly efficient in the overexpression or knockdown of the expression of miR148b-5p (Figure 4(d)). In the next phase, RAW 264.7 cells, stimulated with LPS, were cocultured with hucMSCs transfected with the miR148b-5p inhibitor, miR148b-5p mimics, or negative control for 48 h. QRT-PCR analyses demonstrated that the expression of miR148b-5p was elevated by 3-fold and the expression of 15-lox-1 was reduced by 1-fold in RAW 264.7, cocultured with hucMSCs transfected with mimics but not with inhibitors (Figure 4(e)). It is worth noting that Western blotting showed that overexpression of miR148b-5p lessened the expression of 15-lox-1 protein but the knockdown of miR148b-5p did not exert this peculiarity (Figure 4(f)). These findings proved that miR148b-5p derived from hucMSCs can downregulate the 15-lox-1 expression in macrophages. In this research, we further explored the effect of miR148b-5p on macrophage polarization. It presented that hucMSCs were able to suppress the expression of iNOS (the marker of classically activated (M1) macrophages) and promote the expression of Arg-1 (the representative of alternatively (M2) activated macrophages). Meanwhile, miR148b-5p overexpression in hucMSCs increased the ability to inhibit iNOS and promoted Arg-1 expression. However, the knockdown of miR148b-5p is exactly the opposite (Figure 4(g)). To confirm that miR148b-5p binds directly to 15-lox-1 mRNA, we established luciferase reporters containing the wild type (WT) and mutant type (Mut) of 3′UTR of 15-lox-1 mRNA. In the same manner, the luciferase activity of reporters significantly increased via the miR148b-5p inhibitor, but decreased by miR148b-5p mimics in WT. As has been expected, the luciferase activity of reporters in Mut showed no difference among the three groups (Figure 4(h)). Therefore, we concluded that miR148b-5p from hucMSCs exerts its function to repress the activation of inflammatory macrophages and the expression of 15-lox-1 to attenuate IBD.

### 3.5. miR148b-5p from hucMSCs Attenuates the IBD through Downregulated 15-lox-1 Expression In Vivo

**Figure 5:**
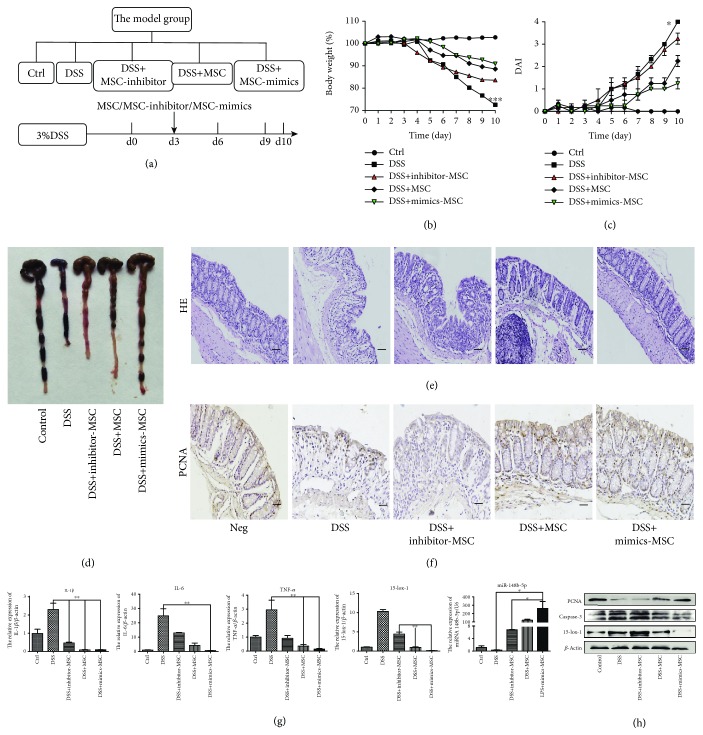
miR148b-5p from hucMSCs attenuates the IBD through downregulated 15-lox-1 expression *in vivo*. (a) The model group was designed to contain the control, DSS, and DSS+MSC transfected with the miR148b-5p inhibitor, negative control, or miR148b-5p mimic groups. (b) The body weight loss and (c) the DAI of each group are presented. (d) The colon lengths of each group are presented. (e) HE of colon tissues of each group is presented (100x, scale bar = 100 *μ*m). (f) IHC of PCNA expression in the colon tissues of each group is presented (100x, scale bar = 100 *μ*m). (g) The inflammatory cytokines (IL-1*β*, IL-6, and TNF-*α*) and miR148b-5p and 15-lox-1 expression of colon tissues were measured via QRT-PCR analyses. (h) The expression of PCNA, caspase-3, 15-lox-1, and *β*-actin proteins in the colon tissues was measured by Western blot. *n* ≥ 6 for each group. Data shown were representative of three independent experiments. Data represent the mean ± SEM. ^∗^ *P* < 0.05, ^∗∗^ *P* < 0.01, and ^∗∗∗^ *P* < 0.001 by ANOVA.

Next, we examined further whether our vitro results generally work in the treatment of IBD. The Bab/c mice were divided to contain the control group, DSS group, and DSS+MSC groups treated with the miR148b-5p inhibitor, negative control, or miR148b-5p mimics (Figure 5(a)). The following were carried out to compare the effects of differently treated hucMSCs on the repair of enteritis. The body weight loss and the DAI of the DSS+MSC+inhibitor group were aggravated compared to those of DSS+MSC group. In sharp contrast to this, hucMSCs transfected with miR148b-5p mimics exerted a stronger function to maintain weight (Figure 5(b)) and reduce the DAI (Figure 5(c)) in IBD mice. In the DSS+MSC+inhibitor group, the colon length was short and the spleen size was as large as that in the DSS group. Nevertheless, miR148b-5p mimics assisted hucMSCs to enhance the colon extension and retain the spleen size in mice with DSS-induced IBD (Figure 5(d) and Figure S5(a)). This difference was more pronounced in HE analysis. The recovery of the intestinal mucosal structure was not much different from that in normal mice, and the formation of splenic nodules was also significantly restored in the DSS+MSC+mimic group. On the contrary, intestinal mucosa tissue was in disorder and splenic nodules were severely damaged in the DSS+MSC+inhibitor group (Figure 5(e) and Figure S5(b)). Immunohistochemistry results showed that hucMSCs transfected with miR148b-5p mimics promoted the proliferation of tissue cells in the intestinal mucosa and spleen, but hucMSCs failed to exhibit this impact when treated with miR148b-5p inhibitors (Figure 5(f) and Figure S5(c)). QRT-PCR analyses further proved that hucMSCs transfected with miR148b-5p mimics obtained a better effect to suppress the expression of inflammatory factor (IL-1*β*, TNF-*α*, and IL-6) and motivate the accumulation of miR148-5p to restrain the expression of 15-lox-1 (Figure 5(g) and Figure S5(d)). Western blot reflected that the expression of 15-lox-1 and caspase-3 decreased observably and the expression of PCNA increased in the colon and spleen tissues in the DSS+MSC+mimic group. Similar to previous results, hucMSCs transfected with the miR148b-5p inhibitor were incapable of inhibiting the apoptosis of inflammatory cells or boosting the proliferation of tissue cells (Figure 5(h) and Figure S5(e)).

Consequently, these results indicate that miR148b-5p from hucMSCs attenuates IBD through downregulating 15-lox-1 expression.

## 4. Discussion

The administration of exogenous mesenchymal stem cells is a promising therapeutic strategy for tissue damage diseases such as liver failure [24], kidney injury [25], rheumatoid arthritis [26], bone disorder [27], and myocardial infarction [28]. In this study, we selected hucMSCs as the novel source of MSCs for IBD therapy in that they can be isolated conveniently and ethically. Several investigations also have demonstrated that hucMSCs could reverse liver failure [29], kidney injury [30], and heart disorders [31]. As we showed in our previous study, the weight loss and bloody stool, identified as IBD symptoms, were relieved and the colon mucosa structure was recovered with the inflammatory response attenuated because of the injection of hucMSCs into DSS-induced IBD mice [20]. However, the mechanism is still unknown. In both UC and CD, the pathogenesis is characterized by the excessive immunoreaction and then the infiltration of inflammatory cells [32]. In the mucosal lesion of the colon, macrophages play an indispensable role and the tissue damage enzymes secreted from them are also involved in the aggravation of inflammatory injury [33]. 15-lox-1, secreted mainly not only by macrophages but also by other tissue cells, is a kind of enzyme leading to bronchial epithelial injury and promoting the lipid signaling pathway induced via linoleic acid and arachidonic acid [17, 34]. In this study, we demonstrated that macrophages were involved in the occurrence and development of enteritis and that hucMSCs could downregulate the expression of 15-lox-1 directly in macrophages. In other words, 15-lox-1 was involved in the process of hucMSCs repairing enteritis.

Several studies have proved that it is paracrine mechanism rather than transdifferentiation that performs most of the functions of MSC immunosuppression [35]. Accumulated evidences indicate that mRNAs and microRNAs can be horizontally transferred via the vesicles, derived from MSCs, to promote tissue injury repair [36, 37]. Xin et al. demonstrated that miRNA-133b could be diverted by MSC-derived exosomes to astrocytes and neurons to boost the nerve regeneration [38, 39]. But few studies have defined specific miRNA involved in the process of MSCs repairing IBD. In this research, we concentrated on the miRNAs silencing the 3′UTR of 15-lox-1 mRNA in macrophages. Qureshi and his colleagues found that miR-148b mimic could elevate the capability of osteogenic linage differentiation of human autologous adipose-derived mesenchymal stem cells (hASCs) [40]. Another team also identified that miR-148b was able to downregulate superoxide production to relieve myocardial infarction [41]. In our study, we found that miR-148b-5p can be complementary with the sequence of the 3′UTR of 15-lox-1 mRNA and hucMSCs transfected with miR148b-5p mimics promoted the proliferation of tissue cells and inhibited the expression of 15-lox-1 in the intestinal mucosa and spleen, but hucMSCs failed to demonstrate this impact when treated with miR148b-5p inhibitors.

A great deal of genetic modification has been applied to boost the immunomodulation function of MSCs in IBD. For example, additional transforming growth factor- (TGF-) 1 expressed in bone marrow mesenchymal stem cells (BMMSCs) derived from miR-21^−/−^ mice was able to promote immunosuppression [42]. The transformation of CD4^+^ and CD8^+^ cytokine generation and the proliferation of MSCs could be realized via IFN-*γ* transfection or IGFBP7 knockdown [43, 44]. In this study, we offer a novel attempt to improve the therapeutic function of hucMSCs through the transfection with miR148b-5p mimics to inhibit the activation and promote phenotypic changes of macrophages. Our results showed that miR148b-5p mimic transfection could enhance the tissue repair capability of hucMSCs in IBD. However, it is necessary to explore in the future whether exosomes derived from hucMSCs transfected with miR148b-5p mimics also would be effective to alleviate the DSS-induced IBD, in that exosomes from MSCs showing great potential for tissue regeneration and to replace stem cell-based therapies. In addition, other miRNAs, silencing the 3′UTR of 15-lox-1 mRNA, also need to be considered in the quest to improve the immune regulatory ability of hucMSCs.

In this study, we first demonstrated that macrophages were involved in DSS-induced IBD and hucMSCs attenuate the IBD by regulating macrophages. Then, we found that hucMSCs regulate the 15-lox-1 expression in macrophages to attenuate the IBD. Eventually, it has been shown that hucMSCs transfected with miR148b-5p mimics could be elevated to promote the tissue repair and inhibit the expression of 15-lox-1 in the intestinal mucosa and spleen, while hucMSCs treated with the miR148b-5p inhibitor failed to perform immunosuppressive function.

## 5. Conclusion

This study reveals that miR148b-5p secreted from hucMSCs attenuates IBD through downregulating the expression of 15-lox-1 in macrophages. These findings provide a novel view for the future research of MSC therapy in IBD or other inflammatory immune diseases.

## Figures and Tables

**Table 1:** Primer sequences for RT-PCR.

Genes	Primer sequence	Annealing temp. (°C)	Amplicon size (bp)
TNF-*α*	FOR: AACTCCAGGCGGTGCCTATG	63	242
	REV: TCCAGCTGCTCCTCCACTTG		
IL-1*β*	FOR: AGCTTCAGGCAGGCAGTATC	61	215
	REV: TCATCTCGGAGCCTGTAGTG		
IL-6	FOR: AAGTCCGGAGAGGAGACTTC	58	487
	REV: TGGATGGTCTTGGTCCTTAG		
15-lox-1	FOR: GGAGGAGGAACTGGAAGAA	66	704
	REV: TCAGAAGATGAGCCTGTAGC		
iNOS	FOR: AGGAGGAGAGAGATCCGATTTAG	62	405
	REV: TCAGACTTCCCTGTCTCAGTAG		
Arg-1	FOR: GAAGAACCCACGGTCTGTGG	60	118
	REV: TCCAACTGCCAGACTGTGGTC		
U6	FOR: CTCGCTTCGGCAGCACA	60	94
	REV: AACGCTTCACGAATTTGCGT		

## Data Availability

All data generated or analyzed during this study are included in this article.

## Abbreviations

MSC: Mesenchymal stem cells
IBD: Inflammatory bowel disease
UC: Ulcerative colitis
CD: Crohn's disease
hucMSCs: Human umbilical cord MSCs
DT: Diphtheria toxin
DSS: Dextran sodium sulfate
LPS: Lipopolysaccharide
Treg: T regulatory
i.p.: Intraperitoneally
i.v.: Intravenously
FCM: Flow cytometry
HE: Hematoxylin-eosin
DAB: Diaminobenzidine
QRTPCR: Quantitative real-time polymerase chain reaction
IL: Interleukin
TNF: Tumor necrosis factor
hASCs: Human autologous adipose-derived mesenchymal stem cells
TGF-1: Transforming growth factor-1
BMMSCs: Bone marrow mesenchymal stem cells.

## Supplementary Materials

Supplementary 1 Supplementary Fig. S1: macrophages are involved in DSS-induced IBD. (A) The size of the spleens of each group is presented. (B) Hematoxylin and eosin staining (HE) of each group is presented (100x, scale bar = 100 *μ*m). (C) The inflammatory cytokines (IL-1*β*, IL-6, and TNF-*α*) and 15-lox-1 expression of spleen tissues were measured via QRT-PCR analyses. *N* ≥ 6 for each group. Data shown were representative of three independent experiments. Data represent the mean ± SEM. ^∗^ *P* < 0.05, ^∗∗^ *P* < 0.01, and ^∗∗∗^ *P* < 0.001 by ANOVA.

Supplementary 2 Supplementary Fig. S2: identification of hucMSCs. (A) FCM was applied to identify the immunophenotype of hucMSCs. (B) Differentiation of hucMSCs towards adipogenic lineages. (C) Differentiation of hucMSCs towards osteogenic lineages.

Supplementary 3 Supplementary Fig. S3: hucMSCs attenuate the DSS-induced IBD through regulating macrophages. (A) The spleens of each group are presented. (B) HE of each group is presented (100x, scale bar = 100 *μ*m). (C) IHC of PCNA expression in the spleen tissues of each group is presented (100x, scale bar = 100 *μ*m). (D) The inflammatory cytokines (IL-1*β*, IL-6, and TNF-*α*) and 15-lox-1 expression of spleen tissues were measured via QRT-PCR analyses. (E) The expression of PCNA, caspase-3, 15-lox-1, and *β*-actin proteins in the spleen tissues was measured by Western blot. *N* ≥ 6 for each group. Data shown were representative of three independent experiments. Data represent the mean ± SEM. ^∗^ *P* < 0.05, ^∗∗^ *P* < 0.01, and ^∗∗∗^ *P* < 0.001 by ANOVA.

Supplementary 4 Supplementary Fig. S4: hucMSCs attenuate the IBD through regulating 15-lox-1 expression in macrophages. (A) The size of the spleens of each group is presented. (B) HE of each group of another model is presented (100x, scale bar = 100 *μ*m). (C) IHC of PCNA expression in the spleen tissues of each group is presented (100x, scale bar = 100 *μ*m). (D) The inflammatory cytokines (IL-1*β*, IL-6, and TNF-*α*) and 15-lox-1 expression of spleen tissues were measured via QRT-PCR analyses. (E) The expression of PCNA, caspase-3, 15-lox-1, and *β*-actin proteins in the spleen tissues was measured by Western blot. *N* ≥ 6 for each group. Data shown were representative of three independent experiments. Data represent the mean ± SEM. ^∗^ *P* < 0.05, ^∗∗^ *P* < 0.01, and ^∗∗∗^ *P* < 0.001 by ANOVA.

Supplementary 5 Supplementary Fig. S5: miR148b-5p from hucMSCs attenuates the IBD through downregulated 15-lox-1 expression in vivo. (A) The size of the spleens of each group was presented. (B) HE of each group is presented (100x, scale bar = 100 *μ*m). (C) IHC of PCNA expression in the spleen tissues of each group is presented (100x, scale bar = 100 *μ*m). (D) The inflammatory cytokines (IL-1*β*, IL-6, and TNF-*α*) and miR148b-5p and 15-lox-1 expression of spleen tissues were measured via QRT-PCR analyses. (E) The expression of PCNA, caspase-3, 15-lox-1, and *β*-actin proteins in the spleen tissues was measured by Western blot. *n* ≥ 6 for each group. Data shown were representative of three independent experiments. Data represent the mean ± SEM. ^∗^ *P* < 0.05, ^∗∗^ *P* < 0.01, and ^∗∗∗^ *P* < 0.001 by ANOVA.
