# Supplementary figures and images for: hucMSCs Attenuate IBD through Releasing miR148b-5p to Inhibit the Expression of 15-lox-1 in Macrophages
Source: Mediators Inflamm. 2019 May 28;2019:6953963. doi: 10.1155/2019/6953963 (PMC6558632; doi:10.1155/2019/6953963)

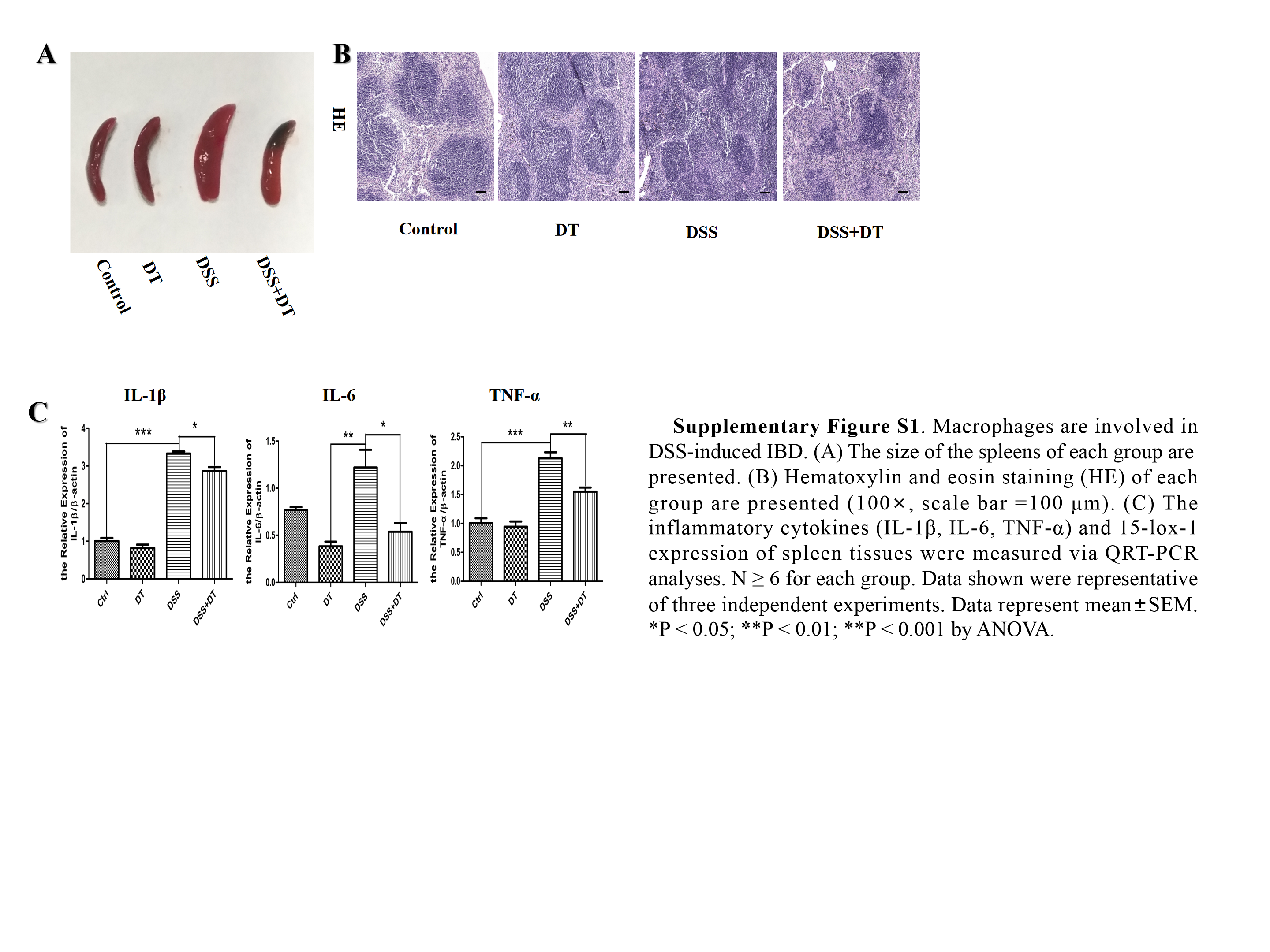

Supplement: Supplementary 1 — Supplementary Fig. S1: macrophages are involved in DSS-induced IBD. (A) The size of the spleens of each group is presented. (B) Hematoxylin and eosin staining (HE) of each group is presented (100x, scale bar = 100 μm). (C) The inflammatory cytokines (IL-1β, IL-6, and TNF-α) and 15-lox-1 expression of spleen tissues were measured via QRT-PCR analyses. N ≥ 6 for each group. Data shown were representative of three independent experiments. Data represent the mean ± SEM. ∗ P < 0.05, ∗∗ P < 0.01, and ∗∗∗ P < 0.001 by ANOVA. [file 6953963.f1.png]

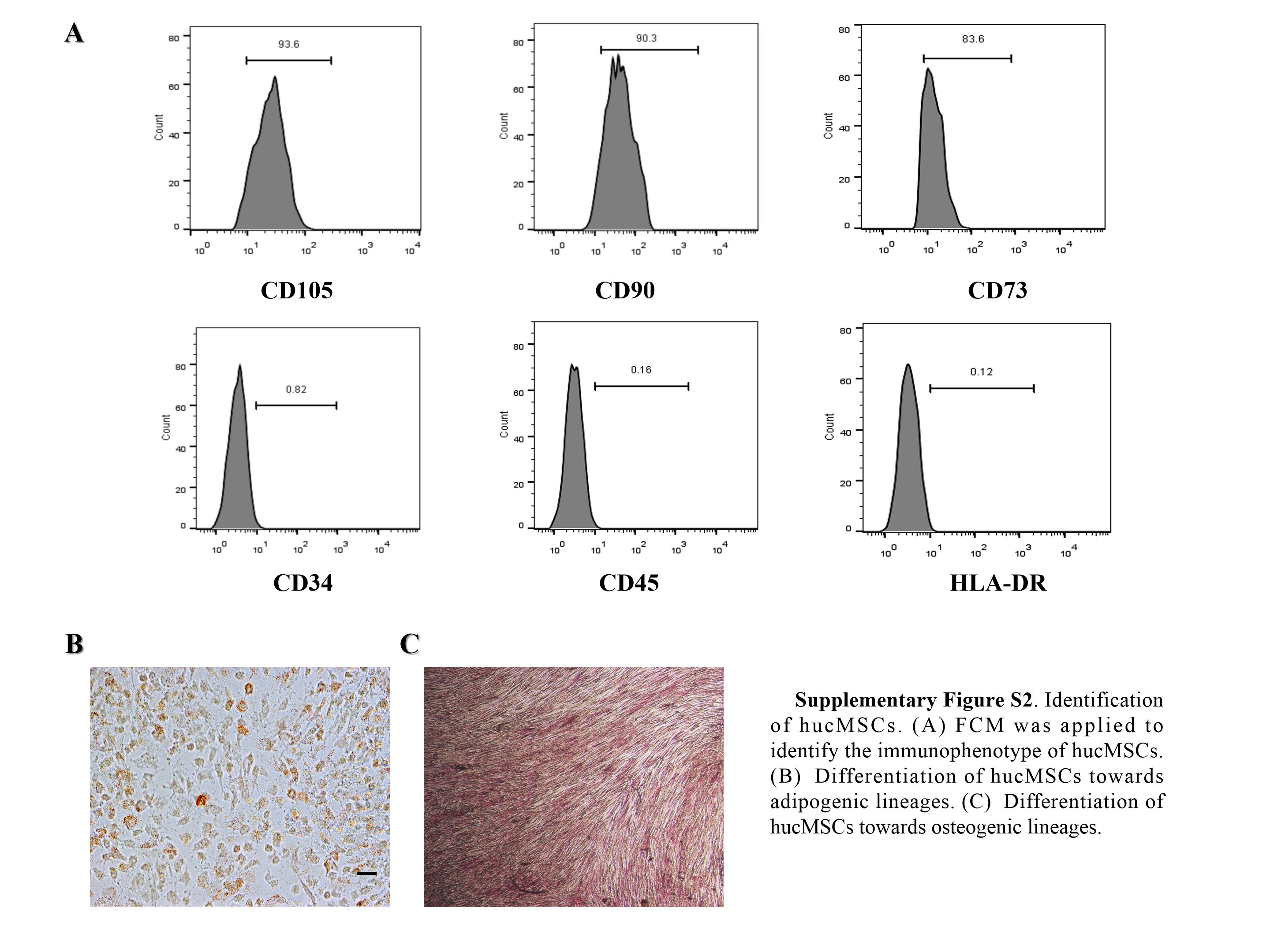

Supplement: Supplementary 2 — Supplementary Fig. S2: identification of hucMSCs. (A) FCM was applied to identify the immunophenotype of hucMSCs. (B) Differentiation of hucMSCs towards adipogenic lineages. (C) Differentiation of hucMSCs towards osteogenic lineages. [file 6953963.f2.png]

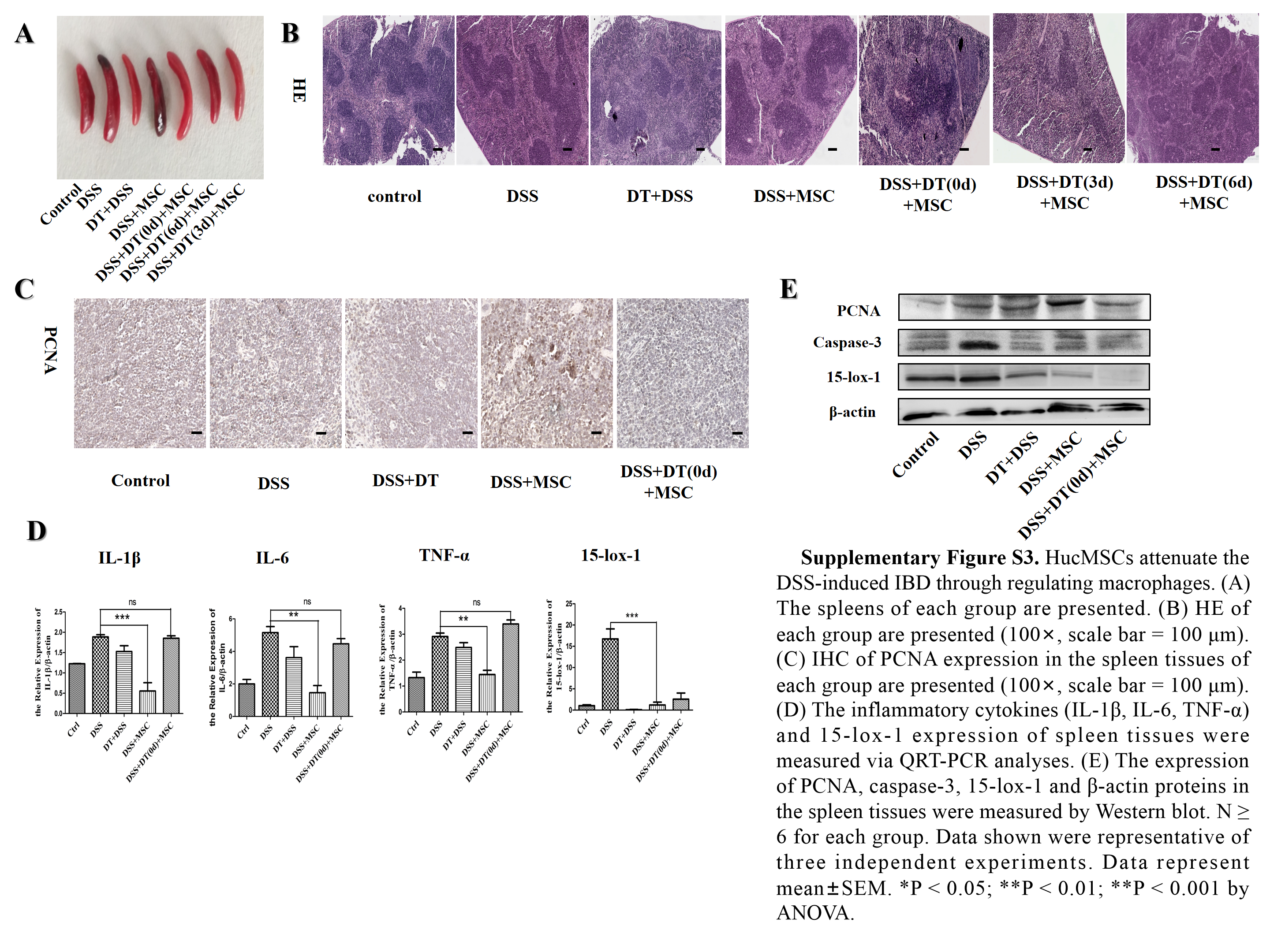

Supplement: Supplementary 3 — Supplementary Fig. S3: hucMSCs attenuate the DSS-induced IBD through regulating macrophages. (A) The spleens of each group are presented. (B) HE of each group is presented (100x, scale bar = 100 μm). (C) IHC of PCNA expression in the spleen tissues of each group is presented (100x, scale bar = 100 μm). (D) The inflammatory cytokines (IL-1β, IL-6, and TNF-α) and 15-lox-1 expression of spleen tissues were measured via QRT-PCR analyses. (E) The expression of PCNA, caspase-3, 15-lox-1, and β-actin proteins in the spleen tissues was measured by Western blot. N ≥ 6 for each group. Data shown were representative of three independent experiments. Data represent the mean ± SEM. ∗ P < 0.05, ∗∗ P < 0.01, and ∗∗∗ P < 0.001 by ANOVA. [file 6953963.f3.png]

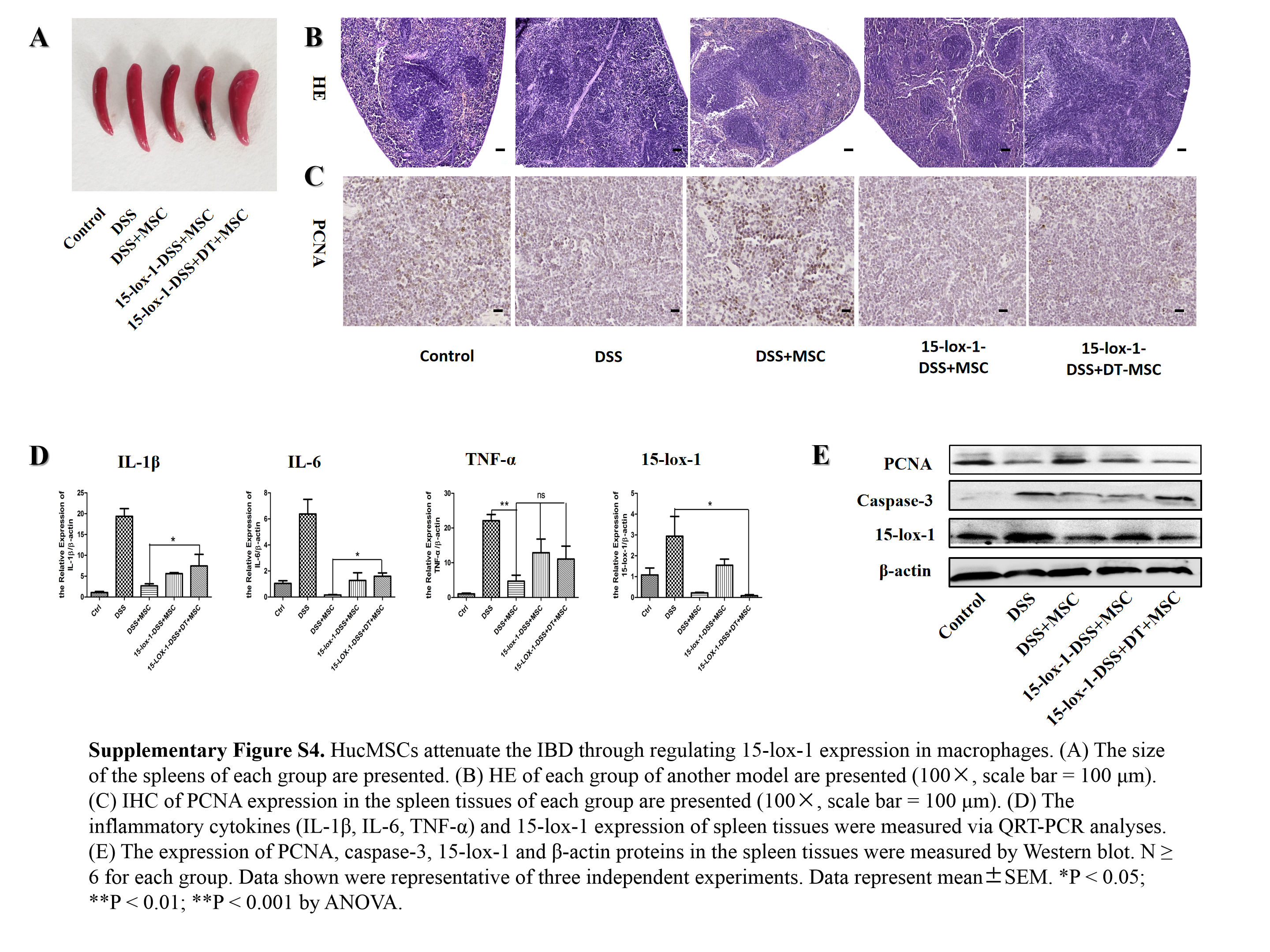

Supplement: Supplementary 4 — Supplementary Fig. S4: hucMSCs attenuate the IBD through regulating 15-lox-1 expression in macrophages. (A) The size of the spleens of each group is presented. (B) HE of each group of another model is presented (100x, scale bar = 100 μm). (C) IHC of PCNA expression in the spleen tissues of each group is presented (100x, scale bar = 100 μm). (D) The inflammatory cytokines (IL-1β, IL-6, and TNF-α) and 15-lox-1 expression of spleen tissues were measured via QRT-PCR analyses. (E) The expression of PCNA, caspase-3, 15-lox-1, and β-actin proteins in the spleen tissues was measured by Western blot. N ≥ 6 for each group. Data shown were representative of three independent experiments. Data represent the mean ± SEM. ∗ P < 0.05, ∗∗ P < 0.01, and ∗∗∗ P < 0.001 by ANOVA. [file 6953963.f4.png]

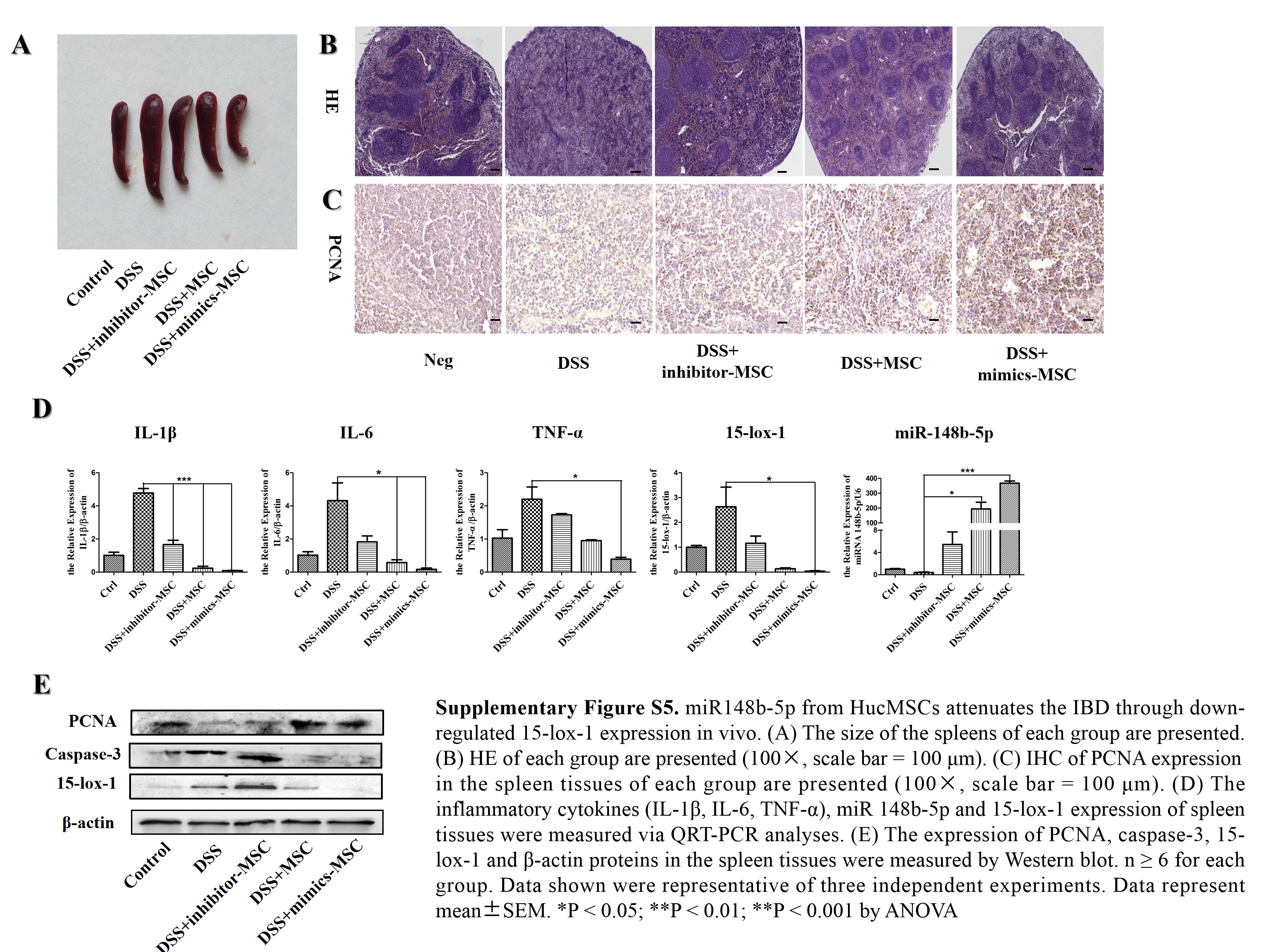

Supplement: Supplementary 5 — Supplementary Fig. S5: miR148b-5p from hucMSCs attenuates the IBD through downregulated 15-lox-1 expression in vivo. (A) The size of the spleens of each group was presented. (B) HE of each group is presented (100x, scale bar = 100 μm). (C) IHC of PCNA expression in the spleen tissues of each group is presented (100x, scale bar = 100 μm). (D) The inflammatory cytokines (IL-1β, IL-6, and TNF-α) and miR148b-5p and 15-lox-1 expression of spleen tissues were measured via QRT-PCR analyses. (E) The expression of PCNA, caspase-3, 15-lox-1, and β-actin proteins in the spleen tissues was measured by Western blot. n ≥ 6 for each group. Data shown were representative of three independent experiments. Data represent the mean ± SEM. ∗ P < 0.05, ∗∗ P < 0.01, and ∗∗∗ P < 0.001 by ANOVA. [file 6953963.f5.png]
